# Design and development of an e-learning patient education program for self-management support in patients with rheumatoid arthritis

**DOI:** 10.1016/j.pecinn.2021.100004

**Published:** 2021-10-31

**Authors:** Line Raunsbæk Knudsen, Kirsten Lomborg, Annette de Thurah

**Affiliations:** a Department of Rheumatology, Aarhus University Hospital, Palle Juul-Jensens Boulevard 59, 8200 Aarhus N, Denmark; b Department of Clinical Medicine, Aarhus University, Palle Juul-Jensens Boulevard 82, 8200 Aarhus N, Denmark; c Steno Diabetes Center Copenhagen, Niels Steensens Vej 2, 2820 Gentofte, Denmark; d Department of Clinical Medicine, University of Copenhagen, Blegdamsvej 3B, 2200 København N, Denmark

**Keywords:** Rheumatoid arthritis, Patient education, Web-based patient education, e-learning, digital health tools, self-management

## Abstract

**Objective:**

To develop an e-learning education program targeting patients with rheumatoid arthritis.

**Methods:**

The development process involved content specification and creative design. It was theoretically framed within theories of multimedia learning and entertainment-education and empirically based on evidence of patient education in rheumatoid arthritis and focus group discussions with stakeholders. We conducted a feasibility test among ten patients to assess the acceptability and usability of the program, and to identify areas to be adjusted.

**Results:**

The following themes for educational needs were found in focus group discussions: *“Knowledge of rheumatoid arthritis,” he disease course and prognosis,” “Medical treatment,” “A new life situation” and “Daily life with rheumatoid arthritis.”* Based on this, an e-learning program covering the disease course, examinations, treatment, and daily life, was created. It combines animations, videos, podcasts, text, speech, and tests. Test persons found the program feasible—that is, clear in content and easy to understand with a suitable pace and coherence between graphics, speech, and text.

**Conclusion:**

This e-learning program is based on solid theoretical knowledge that meets users' needs and is easy to use.

**Innovation:**

This study contributes to the innovation of health care by the development of a new digital tool for patient education.

## Highlights

• Involving stakeholders is essential in the design of digital health tools
• User involvement in the design strengthen the usability of the e-learning programme
• Digital health tools should allow for patients varying needs, abilities and competencies
• Users prefer the use of visual tools over text in the e-learning programme

## 1 Introduction

Rheumatoid arthritis (RA) is a chronic disease that must be managed throughout life depending on disease fluctuations, symptoms, medical treatment, and activities of everyday life [1]. This requires a range of self-management skills, including understanding the disease, managing and reacting appropriately to symptoms, and adjusting to the condition to maintain quality of life [1,2]. According to the European Alliance of Associations for Rheumatology (EULAR), patient education (PE) aims to support patients' knowledge and self-management of their disease [2]. Previous studies have shown that self-management education based on social cognitive theory can promote behavior change, leading to increased self-management of a chronic disease [1,[3], [4], [5]]. Recommendations suggests that PE can be provided as face-to-face or online sessions underpinned by supplementary material, for example, pamphlets and multimedia material [2]. Nevertheless, evidence on how to provide online PE for rheumatic diseases is limited [2].

The World Health Organization (WHO) points out that advances in digital technologies can support healthcare delivery by offering alternatives to standard care [6]. It is also emphasized that digital health facilitates communication and increases patient involvement [6]. Furthermore, digital delivered health information is considered to increase both flexibility and accessibility by using various methods in the presentation of health information [7]. In relation to rheumatic diseases, a survey on the usage of mobile health concluded that although patients had a positive attitude toward mobile health tools, low levels of eHealth literacy emerged [8]. Another study evaluating the quality of websites providing educational contents for patients with RA found that most websites presented accurate information and were user friendly [9]; however, the topics covered were not exhaustive; the reading level was too high; and the use of interactive elements, graphics, and images to support understanding were limited [9]. Likewise, a study examining the readability and suitability of 23 websites of educational material for rheumatic diseases showed that most of the information was designed to have a reading level above the recommended level [10].

Recently, the importance of paying attention to health literacy in the development of multimedia PE tools has been emphasized [11]. In addition, a review of the evidence on telehealth within rheumatic and musculoskeletal disease rehabilitation suggests that the development of telehealth interventions should be based on the involvement of stakeholders, behavioral change theories, and health literacy principles [12]. Hence, several aspects should be considered when creating and using digital health interventions, and despite a range of advantages, challenges remain. In this paper, we describe the design and development process of a web-based e-learning self-management program that targeted patients newly diagnosed with RA. Self-management, self-efficacy, and behavior change theories form the theoretical framework behind the expected effect of the e-learning program. Currently, the program is being tested for efficacy in a randomized controlled trial (RCT) (ClinicalTrials.gov: NCT04669340).

## 2 Material and methods

### 2.1 Study design

**Fig. 1:**
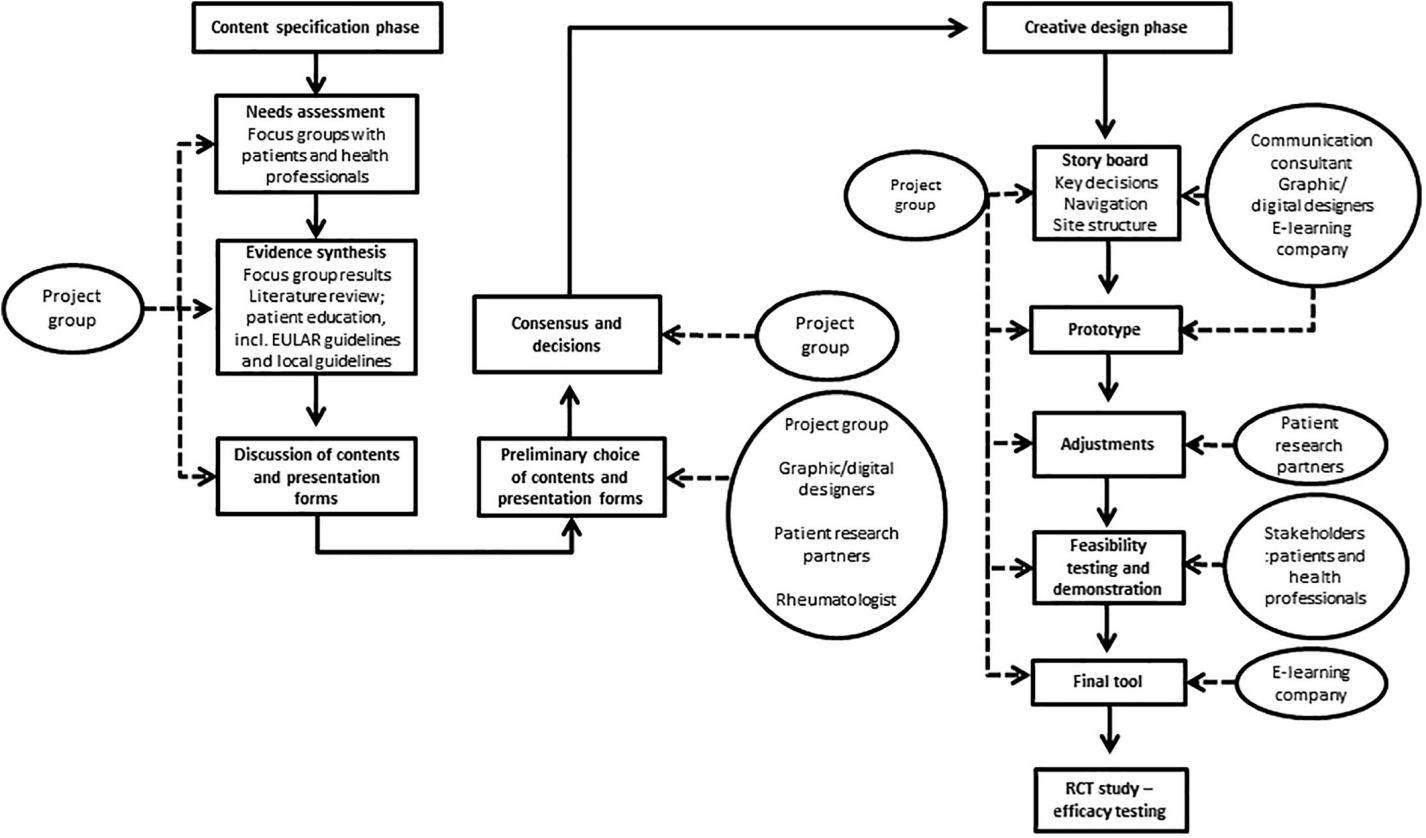
Description of the development process. The figure shows the development of the e-learning program, from the clarifying and content specification phase to the design of the program. The rectangles cover the content of the phases, and the circles illustrate the involvement of collaborators and stakeholders throughout the development.

The development process involved several steps, including a content specification phase involving a systematic literature review and focus group discussions and a creative design phase involving development of the e-learning program following a feasibility test. The program was developed from April 2019 to July 2020 via an iterative process between the primary investigators (LRK, AdT), patient research partners, a communication consultant, digital and graphic designers, and experts on e-learning. We formed a project group comprising the primary investigators (LRK, AdT) and a communication consultant on an ad hoc basis. Further, a rheumatologist and two patient research partners were affiliated with the project to ensure advice on contents, presentation forms, and structure. The technical parts of the project, production and design were carried out by an e-learning company and external consultants, including graphic and digital designers, a software developer, a speaker, a journalist and a communication consultant. The development process is inspired by a systematic process map for the development of web-based decision support interventions [13]. Fig. 1 gives an overview of the development process and project organization.

The study was conducted in accordance with the Helsinki Declaration and the Central Denmark Region Scientific Committee approved the study (no. 1–16–02-52-19). According to Danish Law and the Central Denmark Region Committee of Health Research Ethics, ethical approval is not required for this study. A data processing agreement with the hosting company of the e-learning program is approved by the Technology Transfer Office at Aarhus University. Verbal and written informed consent were obtained from each participant.

### 2.2 Content specification phase: review of the literature and focus groups

Initially, a needs assessment was undertaken. This endeavor included a systematic review of the literature on the educational needs of patients with RA and web-based PE tools followed by qualitative focus group discussions with patients and health professionals (HPs) [14]. The aim of the focus groups was to explore and expand on the educational needs derived from the literature and local guidelines for PE in RA by discussing possible topics in a web-based PE program and generating ideas on how to present contents via multimedia tools.

Three focus groups were established—two for patients and one for HPs—to cover a broad perspective on educational needs for patients with RA and discuss possible topics and presentation forms in a web-based PE program. Data were collected from March to April 2019 in a rheumatology clinic at Aarhus University Hospital, Denmark. Patients were recruited through an online post and handouts in the outpatient clinic and they were selected purposively to achieve diversity regarding sex, age, and disease duration. Eleven patients were included in the study and divided into a group of newly diagnosed patients (diagnosis <1 year) and a group with longer disease durations (>1 year). Inclusion criteria were a diagnosis of RA according to the American College of Rheumatology/European League Against Rheumatism 2010 (ACR/EULAR 2010) criteria [15] and ability to speak, read and understand Danish**.** Two rheumatologists and three nurses with various lengths of employment and levels of experience within rheumatology formed the HP group (Table 1).

**Table 1:** Participants' characteristics in the focus groups and feasibility test.

Focus groups	
Characteristics	Values
Patients	11
Female	9
Male	2
Age, years (mean)	57⁎ (range 35–74)
Disease duration	
<1 year (number of patients)	3
>1 year (number of patients)	9
Years (mean)	9⁎ (range 0.5–30)
Health professionals	5
Rheumatologists	2
Nurses	3
Education, years (mean)	18⁎ (range 2–34)
Experience in rheumatology, years (mean)	13⁎ (range 2–19)

Feasibility test	

Characteristics	Values

Test-persons	10
Female	7
Male	3
Age, years (mean)	58⁎ (range 36–74)
Disease duration, years (mean)	5⁎ (range 0.5–19)

⁎ Values are rounded to the closest whole number.

Focus groups were held for patients and HPs separately and were facilitated by LRK and observed by AdT or a communication consultant to make notes, ask additional questions, and qualify the comprehension of the data. Literature covering educational needs and everyday life with RA [[16], [17], [18], [19]] were used to form a questioning route [20] and the below mentioned activity (appendix A). Questions were composed by LRK and discussed with KL, AdT, and patient research partners to ensure consensus [14]. The questioning route ensured the focus of the discussions; however, a funnel strategy was applied. First, a less structured start was enabled to promote a free discussion, and later, a discussion of specific topics occurred [14]. To ensure a successful transition to the more controlled set of topics [14] and a lively discussion [21], each participant received a bunch of cards illustrating possible topics in PE and modes of presentation in a web-based PE program, for example, via video, animation, or podcast. Participants then prioritized the topics according to their preferences and needs following a joint discussion. Each focus group lasted 90–120 min and were audio recorded, transcribed verbatim and subsequently analyzed inspired by thematic analysis, that is *“a method for identifying, analysing and reporting patterns (themes) within data”* [22]. LRK carried out the analysis; however, ongoing discussions with AdT occurred to ensure agreement on the conclusions. The analysis was organized and managed using QSR International's NVivo 12.2 software [23]. Subsequently, the contents were defined, and the e-learning program was designed.

### 2.3 Creative design phase: didactic considerations and presentation of contents in the e-learning program

Following the focus group discussions, the contents were described in manuscripts written by LRK and AdT and adjusted by the e-learning company. Hereafter, they were adapted to storyboards by graphic and digital designers. In that way, the textual manuscripts were converted into a graphic web-based entity followed by the recording of speech to support the graphical illustrations.

Our e-learning program is based on a program theory inspired by Funnel and Rogers [24], who described how theories of change can explain the drivers by which behavior change occurs in individuals and where theories of action can explain the interventions that can activate these changes. The theoretical framework in the design of the program draws on elements of the cognitive theory of multimedia learning (CTML) [25], entertainment-education (E-E) [26,27] and didactics. This is done in order to promote self-management in patients with RA attending patient education through the e-learning program and allow for different health literacy levels among users.

Structure and presentation of contents in spoken language, consistency, and a common thread in the tone were ensured through the firm design process. In doing this, text-heavy presentations were avoided, and text was applied only to support the spoken words. CTML outlines an active learning process through which people build coherent mental representations from words and images. Thus, the program offers a combination of animations, graphics, videos with patients and HPs, podcasts, written text, and spoken words. The intentions behind E-E are implemented via personal patient stories, podcasts, and interactive quizzes.

### 2.4 Feasibility test

In order to evaluate the acceptability and usability of the e-learning program and its user interface the creative design phase was followed by a feasibility test of the prototype. The final program has been adjusted accordingly.

The prototype was tested from May to July 2020 by 10 patients with RA; five patients recruited from the outpatient clinic and five from the previously established focus groups. Like in the focus groups, the inclusion criteria were a diagnosis of RA and ability to speak, read and understand Danish**.** These test subjects were selected purposively to achieve diversity regarding sex, age, and disease duration (Table 1). They all accomplished the e-learning program at home, followed by a 20–45 min semi-structured individual interview by LRK at the hospital or by telephone. The interview covered the general impression of the program, user perception, comprehensibility of the contents, reader-friendliness, layout/design, length and amount of presentations (appendix B). The interviews were audiotaped, verbatim transcribed, and analyzed in order to identify both positive and less positive perceptions, leading to adjustments. This process was managed using QSR International's NVivo 12.2 software [23].

## 3 Results

### 3.1 Content specification phase: educational needs - a synthesis of the evidence and focus group discussions

PE should address several topics because patients' needs vary from the early disease stages throughout the disease course [2]. Furthermore, studies show that health information and educational needs in patients with RA include disease information, such as symptoms, prognosis, comorbidities, and treatment; exercise and training; and self-management and coping strategies [2,[16], [17], [18], [19],^28^]. In addition, emotional and social support seems important to patients with RA [2,[16], [17], [18], [19],^28^].

The four following themes covering educational needs emerged from the analysis of focus group discussions: *“Knowledge of RA, the disease course, and the prognosis,” “Medical treatment,” “A new life situation,”* and *“Daily life with RA*.” In general, patients and HPs agreed on the prioritization of topics into “need-to-know” and “nice-to-know” information when newly diagnosed with RA. Knowledge of the disease (e.g., etiology, the immune system, and symptoms), medical treatment, examinations, the disease course, expected prognosis, and emotional issues were highly prioritized by all groups. Furthermore, patients with longer disease durations emphasized information on non-pharmacological treatment and vaccines. All groups found aspects of daily life essential for coping with the disease but more on a “nice-to-know” basis. Variation within the theme *“Daily life with RA”* was evident and presumably associated with individual needs and preferences from the perspectives of both patients and HPs. Details on the themes derived in the analysis and illustrative quotes can be found in appendix C.

Different presentation forms were discussed, primarily based on the participants' experiences of using the internet for health information and their perceptions of different digital communication tools. In these discussions, it became evident that preferences differed depending on the content. However, graphics, animations, speech, text, and videos were all acknowledged as relevant presentation forms, and a common preference was to use less text and more graphical elements.

### 3.2 Creative design phase: design of the e-learning program - Contents and user interface

We have created a platform that consists of a navigation site with an overview of the program and individual user progress (appendix D). The e-learning program is divided into the three following learning modules: “*Worth knowing about RA” (Module 1)*, *“Additional information about RA” (Module 2)*, and *“Daily life with RA” (Module 3)*.

The contents of the three modules can be seen in Table 2. Among other things, module 1 contains important “need-to-know” information about medical treatment and possible side effects, blood tests, and how to react in case of disease flares. Hence, for safety reasons, it is made mandatory, and the system is designed so that this must be completed before access is given to modules 2 and 3. In modules 2 and 3, users can move around based on their individual needs and preferences. The duration of the full program is approximately 2 h. Documents for download are available to support users who may benefit from supplemental written material (e.g., because of low functional health literacy). Moreover, quizzes are used to activate the users and test users' knowledge on methotrexate and appropriate reactions in case of disease flares, side effects, or infection.

**Table 2:** Contents and topics covered in the e-learning program.

Contents of the e-learning program		
Module 1 (mandatory) “Worth knowing about rheumatoid arthritis (RA)”	Module 2 (optional) “Additional information about RA”	Module 3 (optional) “Daily life with RA”
Understand your disease ▪ *RA* ▪ *The typical disease course* Medical treatment ▪ *Treatment strategy* ▪ *Methotrexate* ▪ *Written material about treatment* ▪ *Blood tests: routine testing* ▪ *Test your knowledge about methotrexate* Symptoms and side effects ▪ *Symptoms* ▪ *Contact the department of rheumatology or your general practitioner* ▪ *Test your knowledge on appropriate reactions in case of disease flare or side effects* Additional knowledge ▪ *FAQ* ▪ *Patient associations, networks, and links*	Causes of RA ▪ *Possible but unclarified causes (heredity, smoking, previous infection)* Frequent symptoms of rheumatoid arthritis ▪ *Pain* ▪ *Pain management* ▪ *Fatigue* ▪ *Fatigue management* ▪ *Reduced mobility* Other symptoms and possible comorbidities ▪ *Rheumatoid nodules* ▪ *Sjogren's syndrome* ▪ *Bursitis* ▪ *Carpal tunnel syndrome* ▪ *Cardiovascular disease* ▪ *Osteoporosis* Examinations and treatment ▪ *Ultrasound imaging* ▪ *Radiography* ▪ *Blood tests: diagnostic tests* ▪ *Medical treatment* ▪ *Steroids: intra-articular injection and intramuscular injection* ▪ *Vaccines*	New life situation ▪ *Emotional issues* ▪ *Two patient stories: “How to manage the disease in everyday life”* Everyday life and lifestyle ▪ *Relations and social life* ▪ *Education and work* ▪ *Physical activity* ▪ *Sleep and rest* ▪ *Sex and intimacy* ▪ *Diets* ▪ *Patient story: “My day with RA”* ▪ *Travels and medicine* ▪ *Youths with rheumatoid arthritis, including a patient story* ▪ *Pregnancy* Practical information, support, and guidance *Links to further information, e.g. social support from society, physiotherapy, subsidies (medical treatment)*

### 3.3 Feasibility test

In general, test-persons had a positive impression of the e-learning program, and contents were considered relevant and useful. In particular, the mandatory medical information of RA and treatment (Module 1) was emphasized as important information when newly diagnosed. Participants liked the flexibility of the program, including the possibility of moving around in the optional parts. Suitable amounts of information were presented, and the contents were considered clear and easy to understand. Test-persons also found the pace suitable, and coherence between graphics, speech, and text was present. In addition, most appreciated the animations and graphics as entertaining, which created a less serious atmosphere despite the seriousness of their situation. Further, the test-persons were satisfied with the navigation site, user interface, and design (graphics, colors, fonts, and composition of the program), which was described as “calm” and “attractive.”

The feasibility test offered suggestions for improvements/alterations. Some issues were corrected immediately, including small technical issues; minor adjustments in colors; changes in the gallery of characters (e.g. better representation of the male gender); and minor misconceptions in illustrations, text and speech. Other adjustments were discussed with the e-learning company and implemented if possible.

The test-persons held different views on the personal patient stories in the videos and podcasts. Some expressed that they facilitated the personalization of the program; others found them less attractive, and many found them too lengthy. Thus, we decided to make patient stories optional by gathering them in modules 2 and 3. Several test-persons were distracted from automatic links with supplementary information, for example, to other websites or additional documents, and therefore, the automation was removed and this was also made optional. Small adjustments were made in podcasts regarding supporting images and background still photography to support speech and enhance attention.

## 4 Discussion and conclusion

### 4.1 Discussion

The overall focus of our study was to develop a web-based PE program to support self-management in patients with RA. We have designed an e-learning program that allows for complexity in telehealth interventions by incorporating a substantial evidence base and theoretical framework, stakeholder involvement, and the health literacy of the users [12]. The question is how these aspects interact and have contributed to the development process.

First, the evidence based on contents in PE within RA was explored. Overall, the topics included in our PE program (information on RA, the disease course, medical treatment, emotional issues facing a new life situation, and daily life with a chronic disease) are in alignment with previous studies examining the educational needs and preferences of patients with RA [2,[16], [17], [18], [19],^28^].

Second, the theoretical framework in the design of the e-learning program, including multimedia learning theory (CTML [25]) and E-E [26,27] should be mentioned. Based on this, we intended to create a program delivering learning content through various multimedia presentation forms in an entertaining manner. This framework was both acknowledged and questioned in the feasibility test. In general, the test persons found the contents easy to understand, the amount of information and the pace suitable, and the animations and graphics entertaining. It also emerged that visual tools were preferred over text. Thus, the various visual and auditory presentation forms seem to be attractive for and favored by the test-persons. This may be explained by the multimedia principle that people learn more deeply from the combination of words and pictures than words alone, including the relation between the auditory and visual channels for processing information [25]. Further, our test persons found the amount of information suitable in the graphical and spoken parts of the program; however, some found the videos too lengthy. An important factor may be that people have limited capacity for the amount of information to be processed [25]. The feasibility test also showed that the effect of personal patient stories in videos and podcasts could be questioned. We included personal patient stories inspired by the idea of E-E and soap operas by using positive role models to promote healthy behavior [27]. The limited excitement or resistance of this among our test persons may be explained by the fact that this approach is developed in a different cultural context. Although E-E interventions are used in varying ways, such as short messages in media and sometimes based on intuition and creativity, E-E is often used in wider contexts, such as campaigns with detailed message design and purposely incorporated theoretical inputs [27]. Thus, a limitation of the use of E-E in our study could be that the theory was not used in full. For example, E-E suggests that three types of characters are used—positive, negative, and transitional—to portray beneficial behavior from all sides [27]. The positive role model was prominent in our patient stories, and a wider choice of role models could possibly have made a difference as to whether the individual can identify with the role models. However, the resistance among our test-persons may also have been caused by group variations because some needed changes in health behavior and others did not.

Third, several studies and reports suggest that user involvement should be prioritized when developing digital health tools [12,[29], [30], [31], [32], [33]]. Recently, a systematic review evaluating the development process of mobile health (mHealth) applications (apps) within rheumatic and musculoskeletal diseases emphasized that the absence of involving stakeholders could lead to fallible conclusions about users' needs and preferences, as well as contents [30]. An overarching strength of the present study is the thorough, collaborative, and iterative process that has guided the development and the involvement of stakeholders, patient research partners, and experts in digital learning and communication. We think that this may strengthen the usability of the program, the effectiveness of the intervention, and future implementation.

Finally, we intended to develop a program that would accommodate a wide target group with different needs, health literacy levels, and learning skills to improve the usability and acceptability of the program. A major Danish study of health literacy showed that people with chronic conditions had more difficulties in understanding health information and engaging with healthcare providers compared with the general population [34]. Further, low levels of health literacy and education were associated with difficulties in understanding health information [34]. Studies of digital health tools have raised concerns that these technologies may exacerbate inequality in health by developing tools suitable only for people with a certain level of health literacy [7,35]. At the same time, digital tools may strengthen equality by integrating solutions that enhance accessibility [7,35], and a visually attractive user interface and integration of different media may appeal to a broader user base [7]. The importance of eHealth literacy—that is, the ability to seek, find, understand, and appraise health information from electronic sources—in the usage of digital health tools has also been emphasized [8,36]. Seventy-five percent of the Danish population accesses the internet several times daily; however, the rate descends with increasing age [37]. Sixty-four percent of Danish people have used the internet to search for health information, with a higher representation of women and people with higher education [37]. The eHealth literacy levels of the users of our e-learning program were not clarified; however, the test-persons in the feasibility test found the contents of the e-learning program easy to understand, they had positive views of the variety of presentation forms, and they considered the user interface and navigation to be intuitive. Based on these findings, we consider our e-learning program to be a simple and easily accessible tool to handle. This indicates that the technology may appeal to a wide group of people with RA, and therefore, it may strengthen the usability and acceptability of the program, as well as adherence to the intervention. However, further research is required to assess the health literacy and eHealth literacy skills present and needed for users of the e-learning program.

A limitation of this study could relate to the composition of the focus groups, especially the patient groups. Although the patients varied in terms of age, gender, and disease duration, most were women and had a longer disease duration. However, as the agreement on the content to be included in the e-learning program was in alignment with the existing evidence on educational needs in patients with RA, we assume that this factor had a minor impact. Another limitation could be the design of our feasibility study, including the sample size. On this basis, it is not possible to evaluate or determine the effect of the learning outcomes behind the program, and further, this study may benefit from a supplementary survey investigating the acceptability of the e-learning program among a larger group of users.

### 4.2 Innovation

This study contributes to the innovation of health care by the development of a new digital tool for patient education in RA. By this tool, we offer a supplement to conventional modes of delivering health information using multimedia and interactive components in patient education. Furthermore, our study builds knowledge to the innovative design process of digital tools and how to include several perspectives, that is the use of experiential and professional knowledge of patients, health professionals and professionals within communication and digital learning into the design.

### 4.3 Conclusion

Web-based patient education in rheumatic diseases is needed; however, knowledge on the development process of digital tools is limited. Our study is based on evidence, solid theoretical knowledge and collaboration involving end users, healthcare providers, and experts in communication and digital learning. Thus, this study offers a concise survey of possible elements integrated in the development of web-based patient education tools that can guide future development processes.

We have developed an e-learning PE program that allows for varying needs, abilities, and competencies among patients with RA attending patient education. Unanswered questions to be examined are the effectiveness of the e-learning program in improving self-management among patients with RA, the patient experiences of receiving information and guidance through this digital tool, and facilitators and barriers to implementing this new technology in clinical practice. This will be investigated in three future studies among patients with RA treated at five rheumatology clinics in Denmark.

## Funding

This work was supported by a grant from 10.13039/501100004191Novo Nordisk 10.13039/100005930Research Foundation (grant number: NNF 18OC0052886), which had no role in any phase of the study, the writing of the manuscript, or the decision to submit the manuscript for publication.

## Author contributions

All authors were involved in drafting the article and revising it, and all authors have approved the final manuscript before submission.

## Declaration of Competing Interest

None.

## Appendix A **Supplementary data**

Supplementary material 1 Image 1

Supplementary material 2 Image 2
