# Supplementary material for: Design and development of an e-learning patient education program for self-management support in patients with rheumatoid arthritis
Source: PEC Innov. 2021 Oct 31;1:100004. doi: 10.1016/j.pecinn.2021.100004 (PMC10194095; doi:10.1016/j.pecinn.2021.100004)
Supplement: Supplementary file 1 — Supplementary material 1 [file mmc1.docx]

**Appendices**

**Appendix A**

| Focus group discussions: Themes and research questions | |
| --- | --- |
| Theme | **Research question** |
| Thoughts when newly diagnosed | What is top of mind for patients when newly diagnosed?^1^  What is top of mind for health professionals when informing patients who are newly diagnosed?^2^ |
| Support from health professionals | Which aspects are important for patients to discuss with health professionals when newly diagnosed and at later disease stages?^1^  What are the considerations of health professionals when deciding what to discuss with patients who are newly diagnosed and at later disease stages?^2^  What are the experiences of health professionals in terms of patients’ preferred topics to discuss when newly diagnosed and at later disease stages?^2^ |
| Web-based information | What are the experiences of health information seeking on the Web and digital communication tools from the patients’ perspective?^1^  What are the experiences of health information seeking on the Web and digital communication tools from the health professionals’ perspective?^2^ |
| Possible topics in the e-learning program and presentation forms | Participants received a bunch of cards with images and text illustrating possible topics in patient education (e.g., immune system, medical treatment, prognosis, pain, fatigue, physical activity) and presentation forms (animations, graphics, podcasts, videos, text, speech).  They were asked to sort the topics into first and second priorities and think about how they would prefer to receive information on the topics on the web. Subsequently, a joint discussion was conducted. |
| This table presents the overall research questions asked in the focus groups with patients and health professionals. The questioning route contained several additional questions.  ^1^ Patient focus groups  ^2^ Health professional focus groups | |

**Appendix B**

| Feasibility test: Themes and research questions in the interview guide | |
| --- | --- |
| Theme | **Research question** |
| General impression of the e-learning program | How do patients experience the e-learning program, and what are the reflections on receiving information on rheumatoid arthritis in this way? |
| Use of the program | Which elements of the program are most and least important for patients? How much time have they spent on the program, and what is the perception of the time spent? |
| Presentation of contents | How do patients experience the presentation of the contents—that is, comprehensibility, reader-friendliness, and presentation forms (animations, graphics, podcasts, videos, text, speech)? |
| User interface and navigation | What are the perceptions of the user interface and navigation—that is, design, colors, and construction? |
| * The table presents the overall research questions. The interview guide contained several additional questions. | |

Appendix C

| Focus group discussions: Summary of the themes derived in the analysis and selected illustrative quotations | | |
| --- | --- | --- |
| Themes | **Summary of themes** | **Quotations** |
| Knowledge of RA, the disease course, and prognosis  Medical treatment  New life situation  Daily life with RA | Understanding the disease, including the autoimmune reaction and symptoms—in particular, pain, swelling joints, and fatigue—was emphasized by both groups. The period before diagnosis was characterized by uncertainty, resulting in a need for information on prognosis and what to expect of the disease course.  Knowledge of medical treatment, choice of treatment, and future possibilities, as well as alternative medicine, were important topics for most patients. The health professionals supported these reflections; however, they emphasized the importance of clarifying the treatment algorithm, matching expectations, and explaining the treatment target.  The participants described a range of emotional reactions as a result of the diagnosis and emphasized a need to address emotional issues. Patients described relief, frustrations, and worries when diagnosed. Some respondents, both patients and health professionals, emphasized examples of patients struggling with their roles and identity as a consequence of diagnosis. Furthermore, an association with reduced mental resources, depression, and cognitive stress was emphasized by both patients and health professionals. However, the realization and acceptance of a new life situation was also expressed in both groups. This was described as challenging; nevertheless, it was also highlighted that patients have a responsibility for taking action to avoid adopting a passive attitude.  Variation within the theme was evident and presumably associated with the individual needs and preferences of patients. All patients described changes in their everyday life, e.g., in physical activity, work, or social relations. This was primarily caused by pain and fatigue, resulting in reduced mental resources. Especially, worries about work, education, and family were present, and for the young patients, thoughts of the future were emphasized. These perspectives were supported by health professionals as topics frequently discussed with patients. | *“I wondered…, what is the typical disease course and what can I expect in 20* –*30 years.… And with or without medicine, what happens to my body?”* (Woman in her 40s)  *“I know a lot has happened with the treatment over the years and it’s nice to hear that you can have a fairly good life.… However, when you are young and have your whole life before you.… Sometimes I think about, what if I can’t work full time? What can I expect…?”* (Woman in her 30s)  *“Patients often ask why they get arthritis and what it will be like in the long term.”* (Nurse)  *“It's about giving hope.… It’s often possible to live as imagined but with some adaptations.”* (Rheumatologist)  *“I want you to explain something about the effect, dose, side effects, and time aspect, and why it is important to take the medication.… I mean, I didn’t know anything and thought about quitting it [the treatment].*” (Woman in her 50s)  *“I would like to know something about the different treatments and the possibilities if my medicine fails.”* (Man in his 70s)  *“I explain to patients that we have an algorithm that fits a great part of the patients based on years of experience, and further, that we don’t know, in advance, which medicine will be the best for the individual.”* (Rheumatologist)  *“We need to explain the goals of the treatment, such as the prevention of joint destruction.”* (Rheumatologist)    *“I mean,* *I’m young and suddenly my life was upside down.… Normally, I’m very active, but suddenly my joints were painful… and the fatigue.… You are vulnerable when you are unaware of what’s happening and you need to change everyday life.”* (Woman in her 30s)  *“It’s hard to accept that you can’t do things as usual.”* (Woman in her 60s)  *“I think it’s very important to give words to the emotional reactions.”* (Woman in her 50s)  *“It's different.… Some are in a state of shock, especially if they are not prepared on the potential diagnosis…, and others may have had a feeling or the diagnosis has been mentioned, and therefore, they take it step by step.”* (Nurse)  *“Cognitive challenges, lack of mental resources and concentration difficulty are emphasized by some patients.… Fulfilling their role, both socially and in work situation, may be difficult and may cause a feeling of losing identity.”* (Rheumatologist)  *“Sometimes, self-pity is present, and I think it’s deeply unfair; however, I know that I need to shift my perspective and go through it by taking responsibility.… It may be challenging, but by doing so, I'm taking the initiative, because I cannot rely solely on the treatment and the physician’s suggestions.”* (Woman in her 50s)  *“You have to go into that process* [accept the disease]*.… It's important to learn and live with the disease. Actually, I have been better by accepting my life, although I find it limiting.”* (Woman in her 50s).    *“I have always been very active, both professionally and personally…, and then from one day to another—this fatigue and exhaustion.… It’s frustrating. So when I have a good day, I sometimes overdo my activities.”* (Woman in her 40s)  *“It was difficult in the beginning, but I think you need to talk about it [the fatigue], although it can be difficult.…* *In particular, when I am in a new setting where they don’t know me. I also need to rest before social activities or else I fall asleep.… Luckily, my relations are lovely and they support me.”* (Woman in her 30s)  *“Work and education when you are young and there is a long working life ahead.… What if I cannot work that many hours—what are my possibilities?”* (Woman in her 30s)  *“I have a reception job three days a week and I am always dressed up and use make-up.… That can be challenging because they cannot see how I am doing.”* (Woman in her 60s)  *“I often find patients worrying about the future, such as their prognosis, work life, and family life.”* (Rheumatologist)  *“Among patients with a longer disease duration, and if the disease is in a stable phase, then it’s the fatigue, which is hard to manage because it affects their relations, activities and work.”* (Nurse)  *“I think it’s important with the younger patients to tell them that the disease doesn’t prevent them from having children—but we need to know when planning a pregnancy.”* (Rheumatologist) |
