# Supplementary figures and images for: Design and development of an e-learning patient education program for self-management support in patients with rheumatoid arthritis
Source: PEC Innov. 2021 Oct 31;1:100004. doi: 10.1016/j.pecinn.2021.100004 (PMC10194095; doi:10.1016/j.pecinn.2021.100004)

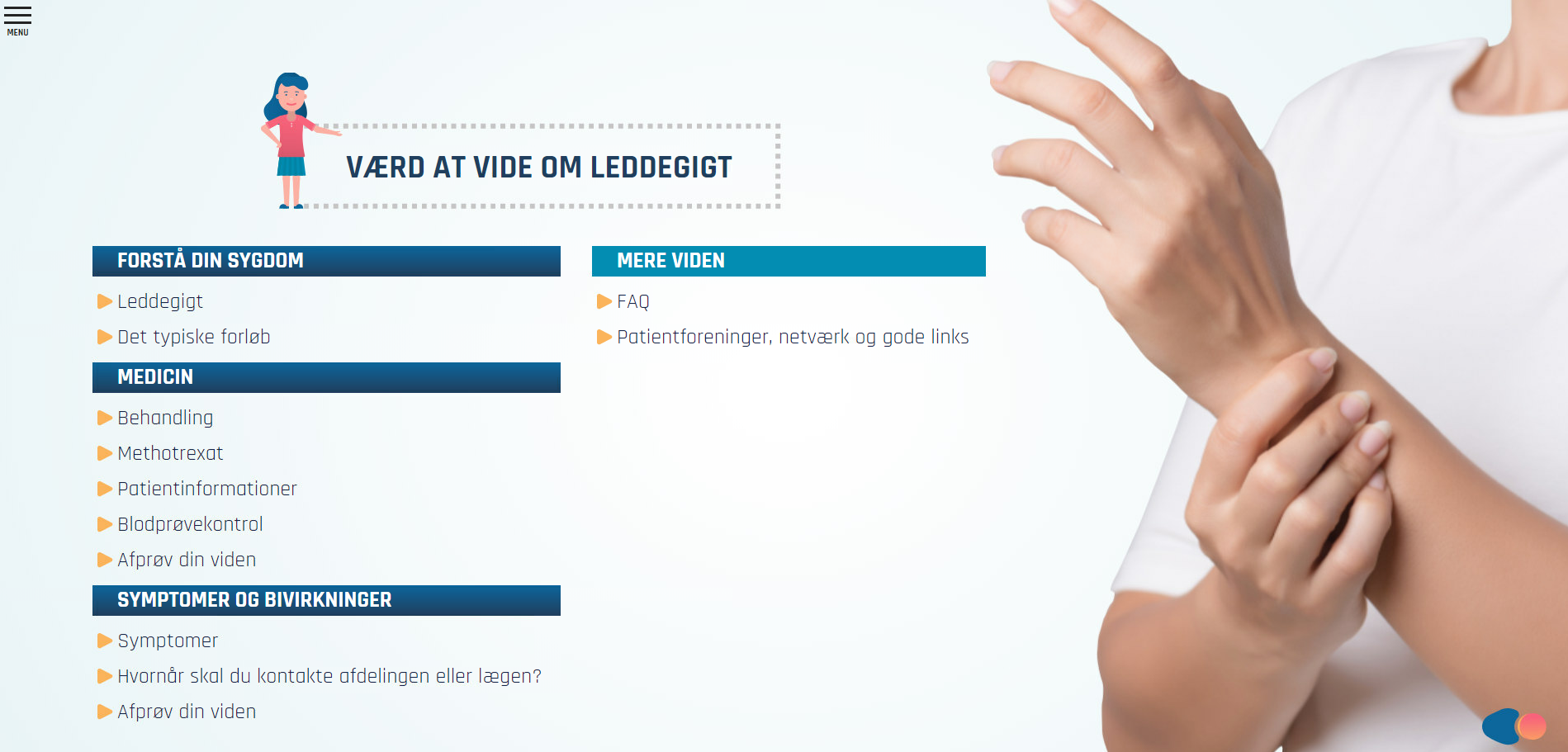

Supplement: Supplementary file 2 — Supplementary material 2 [file mmc2.zip › Appendix D.tif]
